# The relationship of Vascular endothelial growth factor gene polymorphisms and clinical outcome in advanced gastric cancer patients treated with FOLFOX: VEGF polymorphism in gastric cancer

**DOI:** 10.1186/1471-2407-13-43

**Published:** 2013-02-01

**Authors:** Sung Yong Oh, Hyuk-Chan Kwon, Sung Hyun Kim, Suee Lee, Ji Hyun Lee, Jung-Ah Hwang, Seung Hyun Hong, Christian A Graves, Kevin Camphausen, Hyo-Jin Kim, Yeon-Su Lee

**Affiliations:** 1 Department of Internal Medicine, Dong-A University College of Medicine, Busan, Korea; 2 Cancer Genomics Branch, Research Institute, National Cancer Center, Goyang, Gyeonggi-do, 410-769, Korea; 3 Radiation Oncology Branch, National Cancer Institute, Bethesda, Maryland, USA

**Keywords:** VEGF, Polymorphism, Gastric cancer

## Abstract

**Background:**

The aim of this study is to evaluate the associations between vascular endothelial growth factor (VEGF) Single-nucleotide polymorphisms (SNPs) and clinical outcome in advanced gastric cancer patients treated with oxaliplatin, 5-fluorouracil, and leucovorin (FOLFOX).

**Methods:**

Genomic DNA was isolated from whole blood, and six VEGF (−2578C/A, -2489C/T, -1498 T/C, -634 G/C, +936C/T, and +1612 G/A) gene polymorphisms were analyzed by PCR. Levels of serum VEGF were measured using enzyme-linked immunoassays.

**Results:**

Patients with G/G genotype for VEGF -634 G/C gene polymorphism showed a lower response rate (22.2%) than those with G/C or C/C genotype (32.3%, 51.1%; *P* = 0.034). Patients with the VEGF -634 G/C polymorphism G/C + C/C genotype had a longer progression free survival (PFS) of 4.9 months, compared with the PFS of 3.5 months for those with the G/G (*P* = 0.043, log-rank test). By multivariate analysis, this G/G genotype of VEGF -634 G/C polymorphism was identified as an independent prognostic factor (Hazard ratio 1.497, *P* = 0.017).

**Conclusion:**

Our data suggest that G/G genotype of VEGF -634 G/C polymorphism is related to the higher serum levels of VEGF, and poor clinical outcome in advanced gastric cancer patients.

## Background

Gastric cancer remains a significant health problem despite of declining incidence in the West. It is the 4th most common cancer worldwide, accounting for 8.6% of all new cancer diagnoses in 2002 [1]. Although the incidence of stomach cancer among Korean has decreased over the past two decades, gastric cancer is the most common carcinoma in men, and the third most common type of cancer in women as a leading cause of cancer death in Korea [2].

In case of the patients who were most newly diagnosed with gastric cancer or gastric cancer with distant metastasis, the mean 5-year survival rate is recognized to be poor at less than 10% [3]. Up to date, no randomized study on combination chemotherapy has reported a median survival time exceeding 12 months [4]. 5-fluorouracil (5-FU) has been used as a main chemotherapeutic agent for the treatment of gastric cancer, and combination chemotherapy with 5-FU has shown improved clinical outcomes. Even though 5-FU with cisplatin is an effective agent, it has been considered to have a high level of toxicity [4]. Oxaliplatin, another platinum based agent, has a more favorable tolerability profile than cisplatin. The Folinic acid/5-FU/Oxaliplatin combination (FOLFOX) has proven to be an effective first- or second-line treatment agent for advanced gastric cancer [5,6]. However, some patients are predisposed to refractory diseases while others develop resistance after the initial response. Patients may also have a different severity of drug-related adverse events. Increasing demand for improved techniques for the prediction of treatment response and survival may facilitate customized chemotherapy and risk-related therapy, resulting in significantly enhanced survival rates.

Vascular endothelial growth factor (VEGF) is a well known pro-angiogenic growth factor, and its stimulation under hypoxic conditions plays a critical role in promoting the survival of malignant cells in local tumor growth and invasion, and in the development of metastases [7]. Several important roles of VEGF in the progression of human gastric cancer have been reported. The expression of VEGF-A is correlated with tumor vascularity [8], and the frequency of hepatic metastases increased significantly among patients with VEGF positive tumors [9]. The expression of VEGF-A is also correlated with a poor outcome, and is an independent prognostic factor in gastric cancer patients [8,9].

The VEGF gene is located on chromosome 6p21.3, and contains eight exons being separated by seven introns. Several single nucleotide polymorphisms (SNPs) have been described in the VEGF gene some of which have been shown to affect the expression of the gene [10]. Among these SNPs there are five SNPs (−2578 C/A, -1154 G/A, -460 T/C in the VEGF promoter region, +405 G/C in the 5′-untranslated region and +936C/T in the 3′-untranslated region) that are common and are related to VEGF protein synthesis [11]. Very limited amount of published data on VEGF polymorphisms in association with gastric cancer prognosis is available, and the results are diverging [12,13]. These studies show an increased level of association of gastric cancer and/or poor clinical outcomes in the subgroup with genotypes, which would predict a higher level of VEGF expression.

VEGF not only promotes neovascularization and migration but also increases vascular permeability and leakage [14]. This results in an elevated interstitial fluid pressure that prevents effective transport of therapeutic drugs into tumors and thereby, reduces the efficacy of anti-cancer treatment. SNPs in VEGF may alter VEGF protein concentrations, and may relate to inter-individual variation in the risk and progression of selected tumors, and their resistance to treatments. There were few reports that showed the predictive value of VEGF polymorphism to FOLFOX or capecitabine and oxalipatin (XELOX) chemotherapy in colorectal cancer [15,16]. However, no study that has investigated the SNPs of the VEGF gene, and their relationship to the clinical outcomes of gastric cancer patients treated with FOLFOX has yet been published.

The purpose of the present study is to investigate whether VEGF SNPs are associated with clinical outcomes of patients with advanced gastric cancer treated with first-line FOLFOX palliative chemotherapy or not.

## Methods

### Study population

All patients in this study had histologically confirmed adenocarcinoma of the stomach. These patients were treated by FOLFOX chemotherapy. All patients who were in their ages of 18 through 79 had a performance status with a score less than or equal to two according to the Eastern Cooperative Oncology Group scale, and adequate bone marrow as well as renal function Previous adjuvant chemotherapy must be completed at least 6 months before inclusion. Exclusion criteria included the presence of central nervous system metastases, serious or uncontrolled concurrent medical illness, and a history of other malignancies. Written informed consent was obtained from each patient before study entry. The use of all patient materials was approved by the institutional review board of Dong-A University Hospital.

### Patient characteristics

From March 2007 to August 2010, a total of 190 patients enrolled into this study. Demographic details on the patients included in the study are shown in Table 1. The patients consisted of 125 men and 65 women, and their median age was 55 (ranging 24–79). Ninty-seven patients underwent curative operation (stage I, 8; stage II, 28; stage III, 41; stage IV(M0), 20), and a palliative resection was done in 30-stage IV patients. Seventy-nine patients (41.6%) received 5-FU-based adjuvant chemotherapy. Almost all patients had a good performance status. No significant association was detected between the genotypes of the SNPs and patient characteristics (data not shown). Genotyping for the six VEGF polymorphisms were obtained from all 143 patients. The frequencies of each genotype are shown in Table 2.

**Table 1:** Patients’ characteristics

**Variable**	**Subgroup**	**No. of patients**	**%**
Sex	Male	125	65.8
	Female	65	34.2
Age	Median	55 years	
	Range	(24–79 years)	
ECOG performance status	0,1	186	97.9
	2	4	2.1
Lauren	Intestinal	26	13.7
	Diffuse	41	21.6
	Mixed	18	9.5
	Unknown	105	55.3
Initial stage	1	8	4.2
	2	28	14.7
	3	41	21.6
	4	113	59.5
Operation	+	127	66.8
	-	63	33.2
Adjuvant therapy	+	79	41.6
	-	111	58.4
No. of metastasis	1	106	55.8
	2	54	28.4
	> 3	30	15.8
CEA	< 5 ng/ml	119	62.6
	≥ 5 ng/ml	54	28.4
	Unchecked	17	8.9

ECOG: eastern cooperative oncology group, CEA: carcinoembryonic antigen.

**Table 2:** Distribution of genotypes and serum levels of vascular endothelial growth factor

**Genotype**	**Polymorphism**	**No. of patients**	**%**	**Mean ± SD (pg/ml)**	P*
−2578C/A	CC	116	61.1	453.2 ± 278.8	0.606
	CA	63	33.2	520.0 ± 392.3	
	AA	11	5.8	523.9 ± 391.7	
−2489C/T	CC	116	61.1	453.2 ± 278.8	0.117
	CT	60	31.6	478.4 ± 350.0	
	TT	14	7.4	724.0 ± 517.7	
−1498 T/C	TT	116	61.1	453.2 ± 278.8	0.563
	TC	61	32.1	512.2 ± 400.7	
	CC	13	6.8	568.2 ± 324.9	
−634 G/C	GG	54	28.4	889.7 ± 453.7	0.004
	GC	93	48.9	471.4 ± 222.6	
	CC	43	22.6	410.7 ± 222.6	
+936C/T	CC	135	71.1	440.0 ± 292.0	0.722
	CT	45	23.7	495.5 ± 329.3	
	TT	10	5.3	502.6 ± 371.1	
+1612 G/A	GG	139	73.2	472.4 ± 339.2	0.371
	GA	47	24.7	538.3 ± 295.9	
	AA	4	2.1	267.0 ± 159.3	

*by Mann–Whitney.

SD: standard deviation.

### Treatment protocols and dose modification

On day 1, oxaliplatin (85 mg/m^2^) was administered by intravenous (i.v.) infusion in 500 ml of normal saline or dextrose over 2 h. On day 1 and 2, leucovorin (20 mg/m^2^) was administered as an i.v. bolus, immediately followed by 5-FU (400 mg/m^2^) given as a 10-min i.v. bolus, followed by 5-FU (600 mg/m^2^) as a continuous 22-h infusion with a light shield. Dose modifications of oxaliplatin or 5-FU were made for hematologic, gastrointestinal, or neurologic toxic effects on the basis of the most severe grade of toxicity that had occurred during the previous cycle. Treatment could be delayed for up to 2 weeks if symptomatic toxicity persisted, or if the absolute number of neutrophils was < 1,500/μl or platelets count was < 100,000/μl. The 5-FU dose was reduced by 25% for subsequent courses after National Cancer Institute Common Toxicity Criteria (NCI-CTC) grade 3 diarrhea, stomatitis, or dermatitis had occurred. The dose of oxaliplatin was reduced by 25% in subsequent cycles if there were persistent paresthesias between cycles or paresthesias with functional impairment lasting > 7 days. Treatment was continued until there were signs of disease progression, unacceptable toxic effects developed, or the patient refused further treatment.

### Follow-up evaluation and assessment of response

Before each treatment course, a physical examination, routine hematology, biochemistry, and chest X-ray were carried out. Computed tomography scans to define the extent of the disease, and the responses were carried out after four cycles of chemotherapy, or sooner if there was evidence of any clinical deterioration. Patients were assessed before starting each 2-week cycle using the NCI-CTC, except in the case of neurotoxicity. For the neurotoxicity, an oxaliplatin-specific scale was used: grade 1, paresthesias or dysesthesias of short duration, but resolving before the next dosing; grade 2, paresthesias persisting between doses (2 weeks); and grade 3, paresthesias interfering with function.

Responses were evaluated using RECIST criteria. Complete response (CR) was defined as the disappearance of all evidences of disease and the normalization of tumor markers for at least 2 weeks. Partial response (PR) was defined as ≥ 30% reduction in uni-dimensional tumor measurements, without the appearance of any new lesions or the progression of any existing lesion. Progressive disease (PD) was defined as any of the following: 20% increase in the sum of the products of all measurable lesions, appearance of any new lesion, or reappearance of any lesion that had previously disappeared. Stable disease (SD) was defined as a tumor response not fulfilling the criteria for CR, PR, or PD.

### Measurements of serum levels of VEGF

Blood sample was drawn from each participant through venipuncture before chemotherapy and after three cycles of treatment. The blood samples were centrifuged for 10 min at 3,000 r/min at −4°C. The serum was subsequently removed and stored at −80°C until biochemical analysis. Serum VEGF enzyme-linked immunosorbent assay (ELISA) was completed as per manufacturer protocols (R&D Systems, Minneapolis MN). Briefly, serum samples were thawed on wet ice three hours prior to assay. Serum samples were pre-treated with an acidic solution to promote dissociation of VEGF from abundant VEGF binding proteins and stabilized in buffer and preservatives. Samples were plated in 96 well format in duplicate after each of conjugated VEGF-1/HRP polyclonal secondary antibody was added. Substrate solution (H_2_0_2_/tetramethylbenzidine) was then administered for thirty minutes after the reaction was quenched with sulfuric acid. Plates were read at an absorbance of 450 nm on a Victor 3 plate reader (Perkin Elmer, Boston MA). Extrapolated absorbance was analyzed using Masterplex Readerfit ELISA software (Hitachi, Waltham MA) and concentration was determined following a 4 Parameter Logistic curve fit as per manufacturer’s recommendation. Measurements were made by the single investigator blinded to the patients’ clinicopathological data.

### DNA extraction and sample preparation

DNA was extracted from the 75 ul buffy coat using the MagAttract DNA Blood Midi M48 Kit (Qiagen, Inc), using a Qiagen BioRobot M48 workstation, according to the manufacturer’s protocols automatically. The purity and concentration of isolated DNA were determined by Nanodrop® ND-1000 spectrophotometer (Nanodrop technologies, DE, USA). Since we needed more detailed quantity of each sample for genotyping reaction, we measured the quantity of DNA using the Quant-iT™ PicoGreen® dsDNA Assay Kit (Molecular Probes, Inc., USA). We made dry plates for genotyping reaction with 10 ng in each well of 384 plates.

### Candidate polymorphisms and primer design

SNPs were selected from the previous study (11). The six SNPs analyzed were VEGF −2578 C/A SNP (rs699947), VEGF −1498 C/A SNP (rs833061), VEGF −634 G/C SNP (rs2010963), VEGF +936 C/T SNP (rs3025039), and VEGF +1612 G/A SNP (rs10434). The multiplexed assay group was designed to test up to 18 SNPs in the same reaction group using MassARRAY Assay Designer v3.0 (Sequenom, CA).

### Genotyping

Genotyping was carried out using the iPLEX Gold™ assay on the MassARRAY® Platform (Sequenom, CA). PCR reactions were performed in a total volume of 5 ul with 10 ng of genomic DNA, 1.625 mM MgCl_2_, 0.1 unit of HotStarTaq polymerase (Qiagen, Valencia, CA), 0.5 mM dNTP (Invitrogen, Inc.), and 100 nM primers. The PCR reactions started at 94°C for 15 min, followed by 45 cycles at 94°C for 20 s, 50°C for 30 s, and 72°C for 1 min, with the final extension at 72°C for 3 min. Amplified PCR products were treated by SAP mixture in a total 7ul with Shirimp Alkaline Phosphatase enzyme & buffer. SAP reaction started at 37°C for 40 min and 85°C for 5 min. The regions containing target SNP were amplified by PCR and treated by SAP followed by single base extension reaction, resulting in an allele-specific difference in mass between extension products. The extension reactions were performed in a total volume of 9 ul with 50 uM dNTP/dideoxynucleotide phosphate (ddNTP) each, 0.063 unit/ul Thermo Sequenase (both from SEQUENOM, Inc.), and 625 nM to 1.25uM extension primers. Under the cycling conditions, two cycling loops, one of five cycles that sits inside a loop of 40 cycles were used. The sample was denatured at 94°C. Strands are annealed at 52°C for 5 s and extended at 80°C for 5 s. The annealing and extension cycle was repeated four more times for a total of five cycles and then, looped back to the 94°C denaturing step for 5 s. After then, the 5-cycle annealing and extension loop was conducted again. The five annealing and extension steps with the single denaturing step were repeated additional 39 times for a total of 40. The 40 cycles of the 5-cycle annealing and extension steps equate to a total of 200 cycles (5 × 40). A final extension was done at 72°C for three minutes and then, the sample wascooled down up to 4°C. After cleaning up the extension reaction products with SpectroCLEAN, the products were transferred to SpectroCHIP using SpectroPOINT and then, scanned through SpectroREADER (MALDI-TOF). Resulting genotype data were collected by Typer v4.0 (Sequenom, CA).

### Statistical analysis

Serum levels of VEGF were expressed as the means ± standard deviation. Associations between VEGF SNPs and levels of serum VEGF were assessed by Mann–Whitney test. The association between VEGF SNPs and response to chemotherapy was assessed by *χ*^2^ statistics.

The primary end point of the study was to investigate the association between genotypes and progression-free survival (PFS). The PFS and overall survival (OS) were calculated from the date therapy started from the date of disease progression and death, respectively. Patients who were alive at the last follow-up were screened at that time. Patients who were excluded from this study or who died before progression were screened at the time that they were excluded from this study. The association of each marker with survival was analyzed using Kaplan–Meier plots, the log-rank test, and its associated 95% confidence interval (CI) was calculated. Hazard ratios (HRs) for survival, together with their 95% CI, were calculated using Cox proportional hazards regression for age, gender, histological subgroup, performance status, disease stage, and polymorphism subtype.

All tests were two-sided, and *P* < 0.05 was considered statistically significant. Analyses were done using SPSS version 14.0 (SPSS Inc, Chicago, IL).

## Results

### VEGF genotype and chemotherapy response

We analyzed the association of pretreatment serum levels of VEGF with VEGF SNPs. Distribution of VEGF genotypes and its serum levels of VEGF are shown in Table 2. Serum levels of VEGF was significantly higher in carriers of the -634 G/G genotype compared to G/C or C/C (889.7 ± 453.7 vs. 471.4 ± 328.1 vs. 410.7 ± 222.6 pg/ml, respectively, *P* = 0.004). None of the other tested SNPs was associated with serum VEGF level.

The overall chemotherapy response rate for treatment was 34.2% (95% CI: 20.0-40.5%). Six patients achieved complete responses (3.2%), 59 patients achieved partial responses (34.2%), 76 patients showed a stable condition (40.0%) and 49 showed a progressive status (25.8%). Lauren’s classification (*P* = 0.029) and number of metastasis were related to the response to chemotherapy (*P* = 0.034). Other parameters such as gender, age, previous operation, initial stage, adjuvant chemotherapy, and carcinoembryonic antigen (CEA) level were not significantly correlated with the clinical response to FOLFOX chemotherapy. VEGF SNPs and its association with responses are summarized in Table 3. The VEGF-A −634 G/G genotypes were related to inferior response rates compared with G/C or C/C genotypes (22.2%, 32.3%, 51.1%, respectively, *P* = 0.034). None of the other analyzed SNPs predicted a response rate.

**Table 3:** Response according to genotyping of vascular endothelial growth factor

**Genotype**	**Polymorphism**	**ORR**	**%**	P*
−2578C/A	CC	41/116	35.3	0.798
	CA	20/63	31.7	
	AA	3/11	27.3	
−2489C/T	CC	41/116	35.3	0.812
	CT	19/160	31.7	
	TT	4/14	28.6	
−1498 T/C	TT	41/116	30.8	0.832
	TC	19/61	31.1	
	CC	4/13	35.3	
−634 G/C	GG	12/54	22.2	0.034
	GC	30/93	32.3	
	CC	22/43	51.1	
+936C/T	CC	46/135	34.1	0.852
	CT	14/45	31.1	
	TT	4/10	40.0	
+1612 G/A	GG	50/139	36.0	0.333
	GA	12/47	25.5	
	AA	2/4	50.0	

*by Fisher’s exact and chi-square test.

ORR: overall response rate.

### Association of VEGF genotype and survival

**Figure 1:**
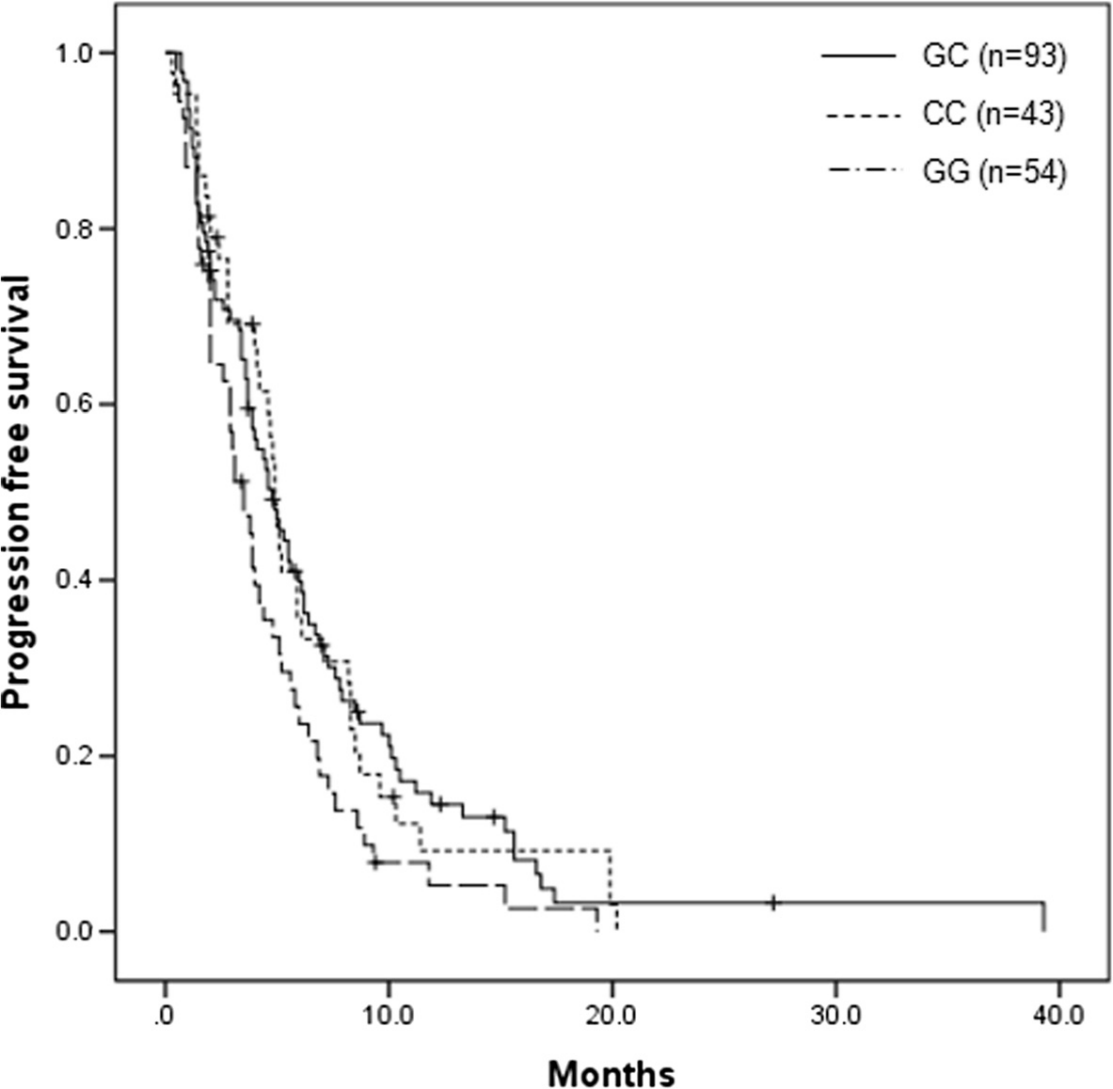
**Kaplan-Meier progression-free survival curve according to vascular endothelial growth factor -634 G/C polymorphisms (*P *= 0.043).**

The median duration of follow-up was 14.6 months (ranging 1.0–48.3 months). The PFS was 4.5 months (95% CI 3.8-5.1 months), and the median OS was 12.9 months (95% CI 10.6-15.2 months). Among clinical parameters evaluated, gender, previous operation, Lauren’s classification, adjuvant chemotherapy, CEA were not correlated with either PFS or OS. Patient’s age was related to both PFS (*P* = 0.035) and OS (*P* = 0.011). Younger patients (less than 60 years of age) had better clinical outcomes. Table 4 shows the association of VEGF SNPs with PFS and OS in the 190 patients analyzed. Patients with the VEGF -634 G/C polymorphism G/C + C/C genotype had a longer PFS of 4.9 months, compared with the PFS of 3.5 months for those with the G/G (*P* = 0.043, Figure 1). No significant influence on OS was observed by the VEGF −634 G/C. However, other VEGF SNPs were not related to PFS, or OS.

**Table 4:** Univariate analysis according to the genotyping of vascular endothelial growth factor

**Genotype**	**Polymorphism**	**No. of patients**	**PFS (Mo)**	P*	**OS (Mo)**	P*
−2578C/A	CC	116	4.9	0.676	12.8	0.423
	CA	63	3.9		14.4	
	AA	11	3.0		11.5	
−2489C/T	CC	116	4.9	0.249	12.8	0.462
	CT	60	4.0		14.4	
	TT	14	2.9		11.5	
−1498 T/C	TT	116	4.9	0.647	12.8	0.440
	TC	61	3.9		13.7	
	CC	13	3.0		11.8	
−634 G/C	GG	54	3.5	0.043	13.1	0.407
	GC	93	4.8		14.4	
	CC	43	4.9		11.5	
+936C/T	CC	135	4.5	0.925	13.1	0.711
	CT	45	4.7		11.9	
	TT	10	3.9		11.9	
+1612 G/A	GG	139	4.4	0.448	12.8	0.644
	GA	47	5.0		14.4	
	AA	4	2.1		10.6	

*by log-rank test.

PFS: progression free survival, Mo: months, OS: overall survival.

Factors that had statistical significance in the univariate models were included in multivariate model. In multivariate analysis, age (hazard ratio (HR): 1.521, 95% CI: 1.105-2.093, P = 0.010), and number of metastasis (HR: 1.375, 95%CI: 1.129-1.674, P = 0.002) remained as independent prognostic factors for PFS. The G/G genotype of -634 G/C polymorphism was also identified as an independent prognostic factor for PFS (HR: 1.497, 95% CI: 1.074-2.088, P = 0.017) (Table 5). No other VEGF SNPs were significant independent prognostic factors impacted on PFS.

**Table 5:** Multivariate analysis

	**Time to progression**		
**Variable**	**HR**	**95% CI**	*p *value*
Age	1.521	1.105–2.093	0.010
Gender	1.313	0.939–1.837	0.111
Performance	2.079	0.743–5.816	0.163
Operation	1.143	0.763–1.713	0.516
Stage	0.941	0.763–1.161	0.571
Lauren type	1.035	0.857–1.250	0.720
Number of metastasis	1.375	1.129–1.674	0.002
−634 G/C polymorphism	1.497	1.074–2.088	0.017

*by cox regression test.

VEGF: vascular endothelial growth factor.

## Discussion

Identification of patients with potentially poor prognosis after FOLFOX chemotherapy would help us to optimize another treatment protocol for patients with advanced gastric cancer. We reported that immunohistochemical staining for Excision Repair Complementation 1 (ERCC1) may be useful in prediction of the clinical outcome in advanced gastric cancer patients treated with modified FOLFOX4 [17]. We have also shown that the Glutathione S-transferase M1 (GSTM1) positive genotype evidenced a significantly better time to progression in cases of advanced gastric cancer being treated with FOLFOX [18].

The association of VEGF gene polymorphisms with the risk or prognosis of gastric cancer has already been shown [12,13]. In a Greek study, 634C/C genotype was significantly associated with increased risk of gastric cancer development, and carrying the -634C/C genotype was associated with decreased overall survival [12]. In a Korean study, +936 T/T genotype had a worse overall survival compared to C/C genotype, and the −460 T/C or C/C genotype was a poor prognostic factor in patients with stage 0 or I gastric cancer [13].

Previous studies have shown that VEGF expression is related to the extent of tumor vascularization and prognosis in solid tumors, and is predictive of resistance to chemotherapy [19]. SNPs in the VEGF gene might influence the delivery of chemotherapy to the cancer cells and may consequently hold predictive information in relation to response [7]. There were several reports of predictive value of VEGF SNPs for bevacizumab treated patients [20-22]. Schultheis *et al.*[20] reported that recurrent ovarian cancer patients with VEGF +937 T polymorphism C/T genotype had a longer PFS when treated with cyclophosphamide and bevacizumab. Schneider *et al*. [21] showed that VEGF -2578AA genotype was associated with a superior median OS, and VEGF -1154A allele also demonstrated a superior median OS in patients with advanced breast cancer with paclitaxel plus bevacizumab treatment. Formica *et al*. [22] reported that VEGF -1154 G/A was an independent prognostic factor for PFS, and VEGF −634 G/C was significantly associated with the response rate in patients with metastatic colorectal cancer patients receiving first-line treatment including fluorouracil, irinotecan, and bevacizumab.

In this study, we assessed six common polymorphisms of the VEGF genes and their association with response and survival in metastatic gastric cancer patients treated with FOLFOX. To our knowledge, this is the first study to demonstrate a relationship between SNPs in the VEGF gene and response to chemotherapy in patients with metastatic gastric cancer. The genotype frequencies of −634 G/C, -2578C/A, or +936C/T in the present study corresponded to those reported in the literature on Korean colorectal cancer patients [23-25], whereas the frequency of the −1498 C/T genotype was similar to that of the Japanese prostate cancer patients [26]. Any minor variation could be explained by sample sizes.

There were two reports that showed the predictive value of VEGF SNPs to FOLFOX or XELOX chemotherapy in colorectal cancer [15,16]. The inferior response rates and shorter PFS were shown in the patients with VEGF −2578 C/A and 405 G/C genotype who were treated with XELOX [15]. Other study showed that VEGF −460 T/C or C/C genotypes were associated with lower response rate to FOLFOX-4 and shorter survival [16]. According to our study, only VEGF -634 G/G genotype showed a significant association with lower response rate and it was translated to short PFS. Shorter overall survival was also shown in Korean colorectal cancer patients with VEGF −634 G/G phenotype [27]. The VEGF −634 G/C likely affects expression at the post-transcriptional level by altering the activity of the internal ribosomal entry site B, thereby enhancing initiation of translation at the AUG start codon and regulating production of the large VEGF isoform, which is translated at an alternative CUG codon [28]. Such changes could be a possible explanation to the low response rates, but several other mechanisms may also be involved. However, we can not specify whether it was the response to 5-FU, oxaliplatin or the combination of both that seemed to be related to SNPs in the VEGF gene or not in this study. None of the rest, of the examined SNPs conferred any clinical significance.

A few studies have reported that VEGF-634 G/C gene polymorphisms are associated with VEGF production. Nonetheless, the results are inconsistent. Awata *et al.*[29] reported that individuals with the −634 C/C genotype had a higher fasting serum VEGF level than those with other genotypes, and that they carried an increased risk of diabetic retinopathy. Meanwhile, Watson *et al.*[10] showed that the −634 G allele is associated with higher VEGF production than the +405C allele. In this study, patients with -634 G/G genotypes were associated with higher circulating VEGF levels.

Kim *et al.* showed that the e VEGF 936 T-allele were associated with inferior survival rates, compared with their corresponding genotypes. However, the VEGF +936 C/T genotype showed no relationship with response to chemotherapy or survival in this study. Difference in disease stages, and sample sizes could probably explain some of these discrepancies.

## Conclusion

To the best of our knowledge, this is the first prospective study that has explored the association between VEGF SNPs and clinical outcomes of metastatic gastric cancer patients treated with FOLFOX chemotherapy. The results demonstrated obvious relationships between genetic variations in the VEGF gene and response to FOLFOX chemotherapy, which translated to a significant difference in PFS.

Irinotecan and taxane-based regimens have been used in the treatment of advanced gastric cancer patients, with a similar survival to those attained with FOLFOX [30,31]. Irinotecan or taxane-based regimens could be the better alternative for patients with VEGF -634 G/G genotype. These findings deserve confirmation in additional prospective studies.

## Competing interests

Above all authors of this paper do not have potential conflicts of interest include employment, consultancies, stock ownership, honoraria, paid expert testimony, patent applications/registrations, and grants or other funding.

## Authors’ contributions

HJA and HSH carried out the molecular genetic studies, OSY drafted the manuscript. OSY, KHC, KSH, LS, and LJH carried out enrolment and treatment of patients. GCA and CK carried out the immunoassay. OSY, LYS and KHC participated in the design of the study and performed the statistical analysis. LYS, KHC and KHJ conceived of the study, and participated in its design and coordination and helped to draft the manuscript. All authors read and approved the final manuscript.

## Pre-publication history

The pre-publication history for this paper can be accessed here:

http://www.biomedcentral.com/1471-2407/13/43/prepub
